# Kinin B_2_ Receptor Mediates Cisplatin-Induced Painful Peripheral Neuropathy by Intracellular Kinase Pathways and TRPA1 Channel Sensitisation

**DOI:** 10.3390/ph16070959

**Published:** 2023-07-04

**Authors:** Gabriela Becker, Maria Fernanda Pessano Fialho, Evelyne Silva Brum, Sara Marchesan Oliveira

**Affiliations:** 1 Laboratory of Neurotoxicity and Psychopharmacology, Federal University of Santa Maria, Santa Maria 97105-900, RS, Brazil; becker.gabi@hotmail.com (G.B.); mariafpessano@outlook.com (M.F.P.F.); esbrum.eb@gmail.com (E.S.B.); 2 Graduate Program in Biological Sciences, Toxicological Biochemistry, Center of Natural and Exact Sciences, Federal University of Santa Maria, Santa Maria 97105-900, RS, Brazil

**Keywords:** neuropathic pain, allodynia, chemotherapy, CIPN, bradykinin, protein kinase Cε

## Abstract

Chemotherapy-induced peripheral neuropathy is a severe clinical problem frequently associated with cisplatin use. Although its pathophysiology is poorly understood, it is known that kinin receptors and the transient receptor potential ankyrin 1 (TRPA1) channel play a significant role in the peripheral neuropathy induced by cisplatin in rodents. However, the role of signalling pathways downstream from B_2_ kinin receptors activation and sensitisation of the TRPA1 channel remains unknown in this model. The cisplatin-induced neuropathy model caused mechanical and cold allodynia in male Swiss mice. Antagonists for kinin B_2_ and B_1_ receptors and the TRPA1 channel attenuated the painful parameters. Local sub-nociceptive doses of kinin B_2_ receptor (bradykinin) and TRPA1 channel (allyl isothiocyanate; AITC) agonists enhanced the painful parameters in cisplatin-treated mice, which their respective antagonists attenuated. Furthermore, we demonstrated the interaction between the kinin B_2_ receptor and the TRPA1 channel in cisplatin-induced peripheral neuropathy since phospholipase C (PLC) and protein kinase C epsilon (PKCε) inhibitors attenuated the increase in mechanical and cold allodynia evoked by bradykinin and AITC in cisplatin-treated mice. Therefore, regulating the activation of signalling pathways downstream from the kinin B_2_ receptors activation and TRPA1 channel sensitisation can mitigate the painful peripheral neuropathy decurrent of the oncology treatment with cisplatin.

## 1. Introduction

Cancer incidence continues to grow worldwide, exerting significant strain on health systems as a major contributor to disease burden [1]. At the same time, early detection and anticancer therapy advances improve cancer patients’ survival rates [2,3]. Chemotherapy, employed for many years, is the cornerstone for treating most forms of cancer, contributing to long-term survival [4,5]. Among chemotherapy drugs, cisplatin, a platinum-based antineoplastic drug, is widely used as a first-line treatment of various solid cancers, including lung, bladder, ovarian, testicular, and head and neck cancer [6,7]. However, its use is associated with neurotoxic effects, collectively referred to as chemotherapy-induced peripheral neuropathy (CIPN) [5,8,9]. Cisplatin-induced peripheral neurotoxicity is due to considerably toxic effects on the peripheral nerves and dorsal root ganglia neurons that are particularly vulnerable to platinum accumulation [8,9,10].

CIPN affects 30–60% of patients in chemotherapy treatment [6,11,12] and can have a prolonged and critical impact on life quality once it is characterised by long-lasting and severe symptoms that affect daily activities and labour capacity [11,13,14]. Clinically, CIPN patients report sensory abnormalities, such as sensory perception changes, burning, numbness, tingling, hypersensitivity to mechanical and thermal stimuli. These symptoms first affect the feet and hands but can also involve proximal regions such as the arms and legs [8,11,13,15,16]. Thus, this clinical situation can make it necessary for dose reduction or even therapy withdrawal, resulting in unsatisfactory cancer treatment and impacting patient survival [7,17].

Currently, there are no effective treatments for alleviating CIPN. Only a moderate recommendation was made for the antidepressant duloxetine by the American Society of Clinical Oncology [18]. This reflects an unclear understanding of the pathophysiology of CIPN. Thus, research to better understand peripheral neuropathy mechanisms is essential for improving the treatment options for this pathological condition.

In this sense, the role of kinin receptors in pain transduction has been widely demonstrated, including in peripheral neuropathy models induced by chemotherapy drugs, such as paclitaxel and vincristine [19,20,21]. Recently, our group also evidenced the involvement of kinin B_2_ and B_1_ receptors in cisplatin-induced painful peripheral neuropathy [22].

Furthermore, the transient potential receptor ankyrin 1 (TRPA1) ion channel is another crucial mediator in pain models, including those induced by chemotherapeutic drugs such as cisplatin [23,24]. TRPA1 is highly expressed in sensory neurons and is co-expressed with kinin receptors [25,26,27,28,29]. In this regard, TRPA1 activity may be modulated by kinin receptors that are G protein-coupled receptors (GPCRs). When activated, these receptors stimulate the phospholipase C (PLC) enzyme generating intracellular mediators such as protein kinase C (PKC) [30,31]. Both PLC and PKC are known to sensitise TRPA1 channel [32,33,34,35]. Furthermore, intracellular sensitisation of the TRPA1 channel can mediate the nociceptive effects of kinins once kinin B_2_ receptor activation increases TRPA1-dependent calcium influx in sensory neurons [34,36].

Based on this, we hypothesised that activation of the kinin B_2_ receptor and its underlying pathways could contribute to TRPA1 sensitisation in the model of painful peripheral neuropathy induced by cisplatin. In this context, using a model of painful peripheral neuropathy induced by cisplatin in mice, we present evidence of the functional interaction between the kinin B_2_ receptor and the TRPA1 channel and its contribution to cisplatin-induced painful symptoms.

## 2. Results

### 2.1. Cisplatin Induces Nociceptive Behaviours in Mice

**Figure 1:**
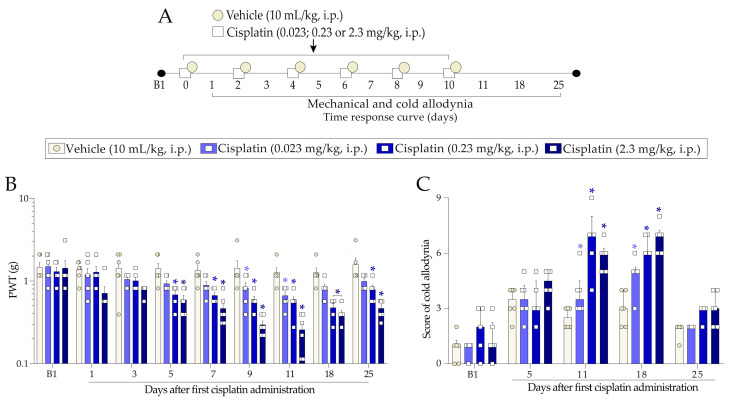
Time and dose-response curve to cisplatin (0.023 mg/kg, 0.23 mg/kg, or 2.3 mg/kg, i.p.) or vehicle (10 mL/kg, i.p.) on mechanical and cold allodynia in mice. Male Swiss mice were treated intraperitoneally (i.p.) with three different doses of cisplatin (0.023, 0.23, and 2.3 mg/kg) or its vehicle (10 mL/kg) every 48 h (days 0, 2, 4, 6, 8, and 10) (**A**). The mechanical PWT of mice was assessed up to 25 days after the first cisplatin dose (**B**). Cold sensitivity was evaluated on alternate days (**C**). Baseline 1 (B1) values were evaluated before the first vehicle or cisplatin dose. The symbols on the bars indicate individual values for each animal. * *p* < 0.05 vs. vehicle group. Data are expressed as the mean + SEM (n = 5–6/group) and were analysed using two-way ANOVA followed by the Bonferroni post-hoc test. PWT: paw withdrawal threshold.

Initially, we characterised the nociceptive profile after three different doses of cisplatin in the peripheral neuropathy experimental model in mice (Figure 1A). Mice treated with cisplatin developed mechanical allodynia, featured by reduction in the mechanical paw withdrawal threshold (PWT) in response to von Frey filament application at a dose of 0.023 mg/kg (at 9 and 11 days after the first cisplatin dose) and doses of 0.23 mg/kg and 2.3 mg/kg (from day 5 up to 25 after the first cisplatin dose) (Figure 1B). Cisplatin at doses of 0.023 and 2.3 mg/kg promoted a maximum reduction of 47 ± 5% and 79 ± 4% of the mechanical threshold 1 day after its last administration (day 11), respectively. Cisplatin at a dose of 0.23 mg/kg promoted a maximum reduction of 69 ± 7% of the mechanical threshold 8 days after its last administration (day 18) (F (24, 152) = 2.89; *p* < 0.0001; Figure 1B). Simultaneously, the three cisplatin doses also increased the mice’s cold sensitivity (from 11 up to 18 days after the first cisplatin dose) concerning the vehicle group (Figure 1C) (F (12, 76) = 4.62; *p* < 0.0001; Figure 1C). These results show that the cisplatin-induced peripheral neuropathy experimental model promoted persistent mechanical and cold allodynia.

Based on the results, we chose the dose of 0.23 mg/kg to continue the study since this dose presented a profile of mechanical and cold allodynia very similar to that of the highest dose of cisplatin used (2.3 mg/kg). In this way, it is possible to investigate the analgesic-like effect of the treatments and possible mechanisms involved in cisplatin-induced neuropathy without causing unnecessary suffering to animals.

### 2.2. Kinin B_2_ and B_1_ Receptors and the TRPA1 Channel Antagonism Alleviate Pain Hypersensitivity Induced by Cisplatin

**Figure 2:**
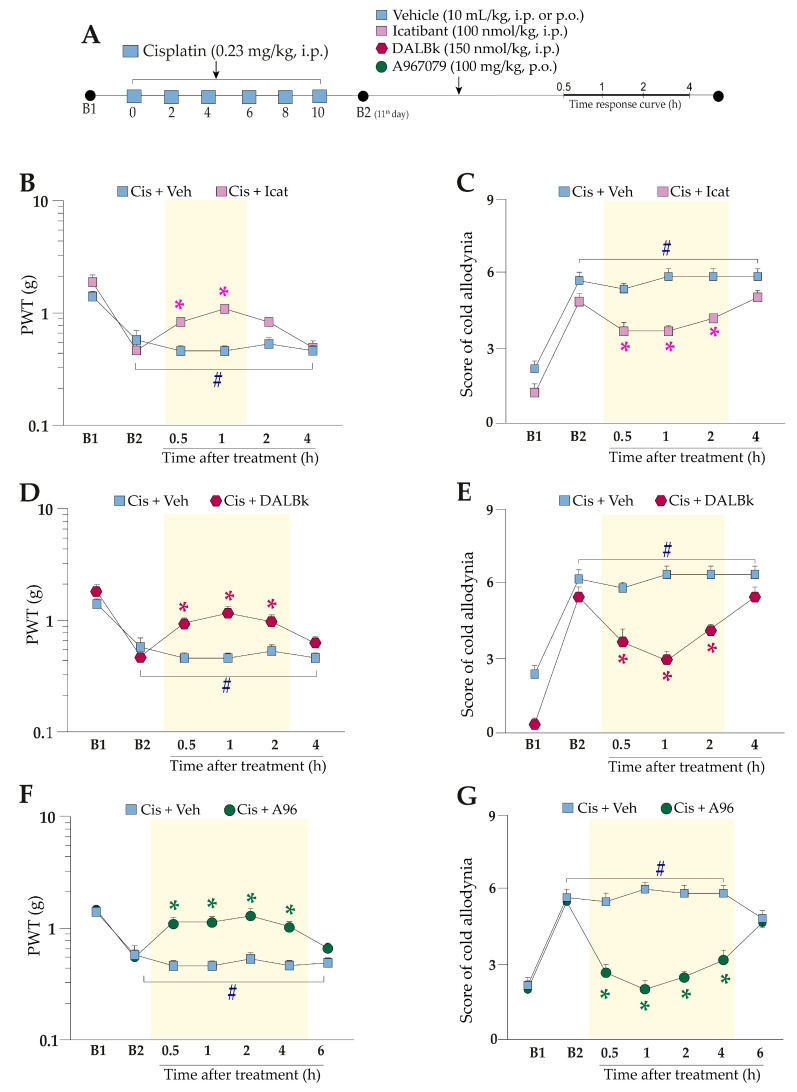
Effects of the antagonists of kinin B_2_ and B_1_ receptors and the TRPA1 channel in peripheral neuropathy induced by cisplatin. Cisplatin (Cis; 0.23 mg/kg) was administered intraperitoneally (i.p.) in mice every 48 h for 10 days. On the 11th day, the animals received a single administration of Icatibant (Icat; 100 nmol/kg, i.p.), DALBk (150 nmol/kg, i.p.), or A967079 (A96; 100 mg/kg, p.o.) or their vehicles (Veh; 10 mL/kg, i.p. and p.o.) (**A**). Mechanical allodynia after treatment with Icatibant, DALBk, or A967079 (**B**,**D**,**F**) and cold allodynia (**C**,**E**,**G**) were again evaluated from 0.5 up to 6 h after antagonist treatments. Baseline 1 (B1) values were measured before the first cisplatin administration. Baseline 2 (B2) values were assessed on the 11th day after the first cisplatin administration and before antagonist treatments. The symbols on the bars indicate individual values for each animal. # *p* < 0.05 vs. B1 values. * *p* < 0.05 vs. cisplatin plus vehicle group. Data are expressed as mean + SEM (n = 6/group) and were analysed using two-way ANOVA followed by the Bonferroni post-hoc test. PWT: paw withdrawal threshold.

We assessed the role of kinin B_2_ and B_1_ receptors and the TRPA1 channel on cisplatin-induced peripheral neuropathy using pharmacological approaches. The antagonists of kinin B_2_ (Icatibant, 100 nmol/kg, i.p.) and B_1_ (DALBk, 100 nmol/kg, i.p.) receptors reduced the mechanical allodynia caused by cisplatin from 0.5 h up to 1 h and 0.5 h up to 2 h after their administrations, with reductions of 67 ± 10% and 74 ± 18% at 1 h after treatments, respectively (Figure 2B,D) (F (5, 50) = 4.93; *p* = 0.001; Figure 2B), (F (5, 50) = 4.78; *p* = 0.0012; Figure 2D). TRPA1 channel antagonist (A967079, 100 mg/kg, p.o.) reduced the mechanical allodynia induced by cisplatin from 0.5 h up to 4 h after its administration (Figure 2F), with a reduction of 86 ± 23% at 2 h after treatment (F (6, 60) = 6.65; *p* < 0.0001; Figure 2F).

The treatment with kinin B_2_ and B_1_ receptor antagonists, Icatibant and DALBK, attenuated the cold allodynia induced by cisplatin administration from 0.5 h up to 2 h after their administration with reductions of 50 ± 5% and 86 ± 9% at 1 h after treatments, respectively (Figure 2C,E) (F (5, 50) = 46.58; *p* < 0.0001 for time, and F (1, 10) = 54.32; *p* < 0.0001 for treatment; Figure 2C), (F (5, 50) = 5.23; *p* = 0.0006; Figure 2E). A967079 also reduced the cold allodynia induced by cisplatin from 0.5 h up to 4 h after its administration (Figure 2G), with a 97 ± 9% reduction at 1 h after the treatment (F (6, 60) = 18.30; *p* < 0.0001; Figure 2G).

**Figure 3:**
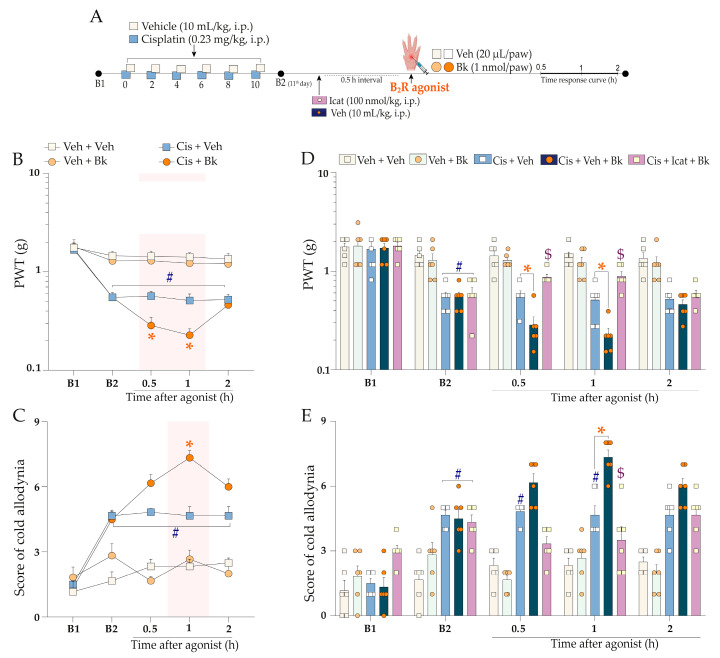
Mechanical and cold allodynia increase evoked by a local sub-nociceptive dose of Bk in mice treated with cisplatin. Cisplatin (Cis; 0.23 mg/kg) or vehicle (Veh; 10 mL/kg) were administered intraperitoneally (i.p.) in mice every 48 h for 10 days. On the 11th day, the animals were injected via the intraplantar route (i.pl.) with a sub-nociceptive dose of kinin B_2_ receptor agonist (Bk; 1 nmol/paw, i.pl.) or vehicle (Veh; 20 µL/paw, i.pl.). Another group of animals, after the last cisplatin dose, received a single administration of Icatibant (Icat; 100 nmol/kg, i.p.) or vehicle (Veh; 10 mL/kg, i.p.), and after 0.5 h they received Bk (1 nmol/paw, i.pl.) or vehicle (Veh; 20 µL/paw, i.pl) using intraplantar injection (**A**). Mechanical PWT (**B**,**D**) and cold allodynia (**C**,**E**) were evaluated from 0.5 h up to 2 h after injection of the agonists. Baseline 1 (B1) values were assessed before cisplatin or vehicle administration. Baseline 2 (B2) values were evaluated on the 11th day after the first cisplatin or vehicle dose and before the antagonist treatment. The symbols on the bars indicate individual values for each animal. ^#^ *p* < 0.05 vs. vehicle plus vehicle group. * *p* < 0.05 vs. cisplatin plus vehicle group. ^$^ *p* < 0.05 and vs. cisplatin plus Bk. Data are expressed as mean + SEM (n = 6/group) and were analysed using two-way ANOVA followed by the Bonferroni post-hoc test. PWT: paw withdrawal threshold. B_2_R: kinin B_2_ receptor.

Subsequently, we evaluated nociceptive behaviours after intraplantar injection of sub-nociceptive doses of agonists of kinin B_2_ and B_1_ receptors and TRPA1 channel in the experimental model of cisplatin-induced peripheral neuropathy. Initially, mice treated with cisplatin presented mechanical and cold allodynia compared to the vehicle group. Bradykinin (Bk; 1 nmol/paw), injected using the intraplantar (i.pl.) route (Figure 3A), enhanced mechanical nociception from 0.5 h up to 1 h and cold sensitivity at 1 h after its administration in animals previously treated with cisplatin when compared to the cisplatin plus vehicle group (Figure 3B,C) (F (12, 80) = 6.57; *p* < 0.0001; Figure 3B), (F (12, 80) = 9.87; *p* < 0.0001; Figure 3C). As expected, the sub-nociceptive dose of Bk did not alter the mechanical or cold sensitivity in animals previously treated with the vehicle (Figure 3B,C). The kinin B_2_ receptor antagonist, Icatibant (100 nmol/kg, i.p.), attenuated the enhancement of Bk-induced mechanical nociception from 0.5 h up to 1 h after intraplantar Bk injection, with an inhibition of 44 ± 6% at 1 h (Figure 3D) (F (16, 100) = 6.14; *p* < 0.0001; Figure 3D). Icatibant also prevented the increase in Bk-evoked cold sensitivity in cisplatin-treated animals, with inhibition of 64 ± 17% at 1 h (Figure 3E) (F (16, 100) = 8.35; *p* < 0.0001; Figure 3E).

**Figure 4:**
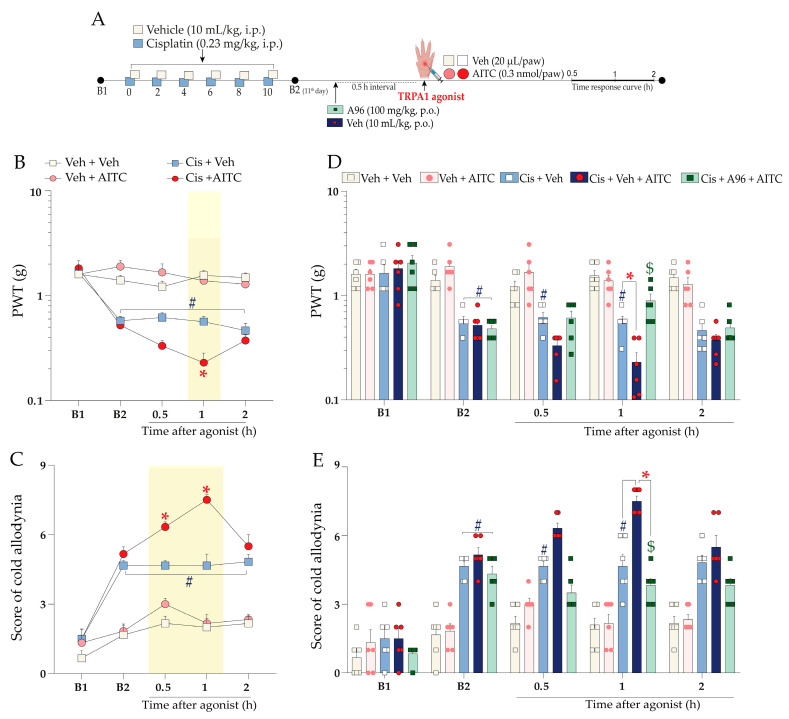
Mechanical and cold allodynia increase evoked by a local sub-nociceptive dose of AITC in mice treated with cisplatin. Cisplatin (Cis; 0.23 mg/kg) or vehicle (Veh; 10 mL/kg) were administered intraperitoneally (i.p.) in mice every 48 h for 10 days. On the 11th day, the animals were injected via the intraplantar route (i.pl.) with a sub-nociceptive dose of TRPA1 agonist (AITC; 0.3 nmol/paw, i.pl.) or vehicle (Veh; 20 µL/paw, i.pl.). Another group of animals, after the last cisplatin dose, received a single administration of A967079 (A96; 100 mg/kg, p.o.) or vehicle (Veh; 10 mL/kg, p.o.), and after 0.5 h they received the AITC (0.3 nmol/paw, i.pl.) or vehicle (Veh; 20 µL/paw, i.pl) via intraplantar injection (**A**). Mechanical PWT (**B**,**D**) and cold allodynia (**C**,**E**) were evaluated from 0.5 h up to 2 h after injection of the agonists. Baseline 1 (B1) values were assessed before cisplatin or vehicle administration. Baseline 2 (B2) values were measured on the 11th day after the first cisplatin or vehicle dose and before antagonist treatment. The symbols on the bars indicate individual values for each animal. ^#^ *p* < 0.05 vs. vehicle plus vehicle group. * *p* < 0.05 vs. cisplatin plus vehicle group. ^$^ *p* < 0.05 vs. cisplatin plus AITC. Data are expressed as mean + SEM (n = 6/group) and were analysed using two-way ANOVA followed by the Bonferroni post-hoc test. PWT: paw withdrawal threshold.

The intraplantar injection of the sub-nociceptive dose of TRPA1 agonist allyl isothiocyanate (AITC; 0.3 nmol/paw) (Figure 4A) enhanced the mechanical nociception at 1 h and cold sensitivity from 0.5 h up to 1 h after its injection in cisplatin-treated mice compared to the cisplatin plus vehicle group (Figure 4B,C) (F (12, 80) = 12.05; *p* < 0.0001; Figure 4B), (F (12, 80) = 6.30; *p* < 0.0001; Figure 4C). TRPA1 antagonist A967079 (100 mg/kg, p.o.) prevented the enhancement of AITC-evoked mechanical nociception and cold sensitivity, with inhibition of 54 ± 7% and 90 ± 7% at 1 h after intraplantar AITC injection, respectively (Figure 4D,E) (F (16, 100) = 9.34; *p* < 0.0001; Figure 4D), (F (16, 100) = 5.80; *p* < 0.0001; Figure 4E). The mechanical or cold sensitivity in mice previously treated with vehicle did not change after a sub-nociceptive dose of AITC (Figure 4B,C).

The sub-nociceptive dose of the kinin B_1_ receptor agonist DABk (3 nmol/paw, i.pl.) did not enhance nociceptive behaviours (mechanical and cold allodynia) in mice previously treated with cisplatin. Thus, the remainder of this study focused on the kinin B_2_ receptor and TRPA1 channel.

### 2.3. Functional Interaction of Kinin B_2_ Receptor and TRPA1 Channel Cooperate to Cisplatin-Induced Painful Peripheral Neuropathy

**Figure 5:**
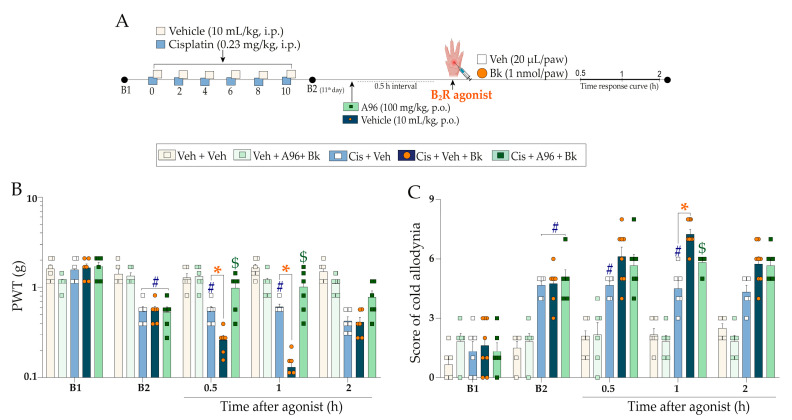
TRPA1 channel contributes to nociceptive behaviours increase induced by a sub-nociceptive dose of Bk in cisplatin-treated mice. Cisplatin (Cis; 0.23 mg/kg) or vehicle (Veh; 10 mL/kg) were administered intraperitoneally (i.p.) in mice every 48 h for 10 days. On the 11th day, a single dose of A967079 (A96; 100 mg/kg, p.o.) or vehicle (Veh; 10 mL/kg, p.o.) was administered. After 0.5 h, the animals received intraplantar (i.pl.) injection of Bk (1 nmol/paw, i.pl.) or vehicle (Veh; 20 μL/paw, i.pl.) (**A**). The mechanical PWT (**B**) and cold allodynia (**C**) were measured from 0.5 h up to 2 h after injection of the agonist. Baseline 1 (B1) values were evaluated before cisplatin or vehicle administration. Baseline 2 (B2) values were measured on the 11th day after the first cisplatin or vehicle dose and before antagonist treatment. The symbols on the bars indicate individual values for each animal. ^#^ *p* < 0.05 vs. Vehicle plus vehicle group. * *p* < 0.05 vs. cisplatin plus vehicle group. ^$^ *p* < 0.05 vs. cisplatin plus Bk. Data are expressed as mean + SEM (n = 6–8/group) and were analysed using two-way ANOVA followed by the Bonferroni post-hoc test. PWT: paw withdrawal threshold. B_2_R: kinin B_2_ receptor.

The sub-nociceptive dose of the kinin B_2_ receptor agonist, Bk (1 nmol/paw, i.pl.) (Figure 5A) increased mechanical nociception from 0.5 h up to 1 h and cold sensitivity at 1 h after its intraplantar injection in animals previously treated with cisplatin. These Bk-evoked effects were alleviated using pre-treatment with TRPA1 channel antagonist, A967079 (100 mg/kg, p.o.) with maximum inhibitions of 57 ± 13% to mechanical allodynia at 1 h and 25 ± 3% to cold allodynia (Figure 5B,C) (F (16, 100) = 14.32; *p* < 0.0001; Figure 5B), (F (16, 108) = 6.31; *p* < 0.0001; Figure 5C).

**Figure 6:**
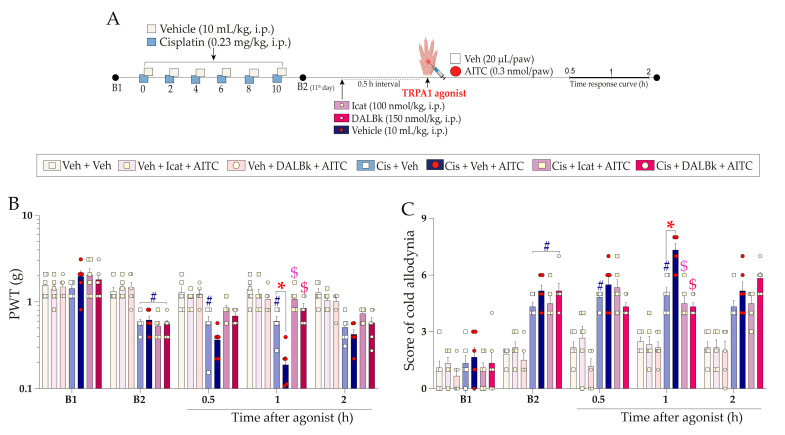
Kinin receptors cooperate with nociceptive behaviours increase induced by a sub-nociceptive dose of TRPA1 agonist in cisplatin-treated mice. Cisplatin (Cis; 0.23 mg/kg) or vehicle (Veh; 10 mL/kg) were administered intraperitoneally (i.p.) in mice every 48 h for 10 days. On the 11th day, Icatibant (Icat; 100 nmol/kg, i.p.), or DALBk (150 nmol/kg, i.p.), or vehicle (Veh; 10 mL/kg, i.p.) were administered. After 0.5 h, AITC (0.3 nmol/paw, intraplantar; i.pl.) or vehicle (Veh; 20 μL/paw, i.pl.) were injected via intraplantar route (**A**). The mechanical PWT (**B**) and cold allodynia (**C**) were evaluated from 0.5 h up to 2 h after injection of the agonist. Baseline 1 (B1) values were assessed before cisplatin or vehicle administration. Baseline 2 (B2) values were measured on the 11th day after the first cisplatin or vehicle dose and before the treatments. The symbols on the bars indicate individual values for each animal. ^#^ *p* < 0.05 vs. vehicle plus vehicle group. * *p* < 0.05 vs. cisplatin plus vehicle group. ^$^ *p* < 0.05 vs. cisplatin plus AITC. Data are expressed as the mean + SEM (n = 6/group) and were analysed using two-way ANOVA followed by the Bonferroni post-hoc test. PWT: paw withdrawal threshold.

Likewise, we evaluated the potential of kinin B_2_ and B_1_ receptor antagonists in preventing the sensitizing effect induced by the TRPA1 agonist (Figure 6A). In a sub-nociceptive dose, local AITC (0.3 nmol/paw, i.pl.) enhanced mechanical nociception and cold sensitivity at 1 h after intraplantar injection in mice treated with cisplatin compared to the cisplatin plus vehicle group (Figure 6B,C) (F (24, 140) = 7.29; *p* < 0.0001; Figure 6B), (F (24, 140) = 5.01; *p* < 0.0001; Figure 6C). The kinin B_2_ receptor antagonist, Icatibant (100 nmol/kg, i.p.) and kinin B_1_ receptor antagonist, DALBk (150 nmol/kg, i.p.) prevented enhancement of the AITC-evoked mechanical and cold sensitivity in animals previously treated with cisplatin with inhibitions of 57 ± 13% and 25 ± 3%, respectively (Figure 6B,C).

### 2.4. The Molecular Signalling Mechanisms Downstream of kinin B_2_ Receptor Activation Contribute to Sensitising the TRPA1 Channel in Mice Treated with Cisplatin

**Figure 7:**
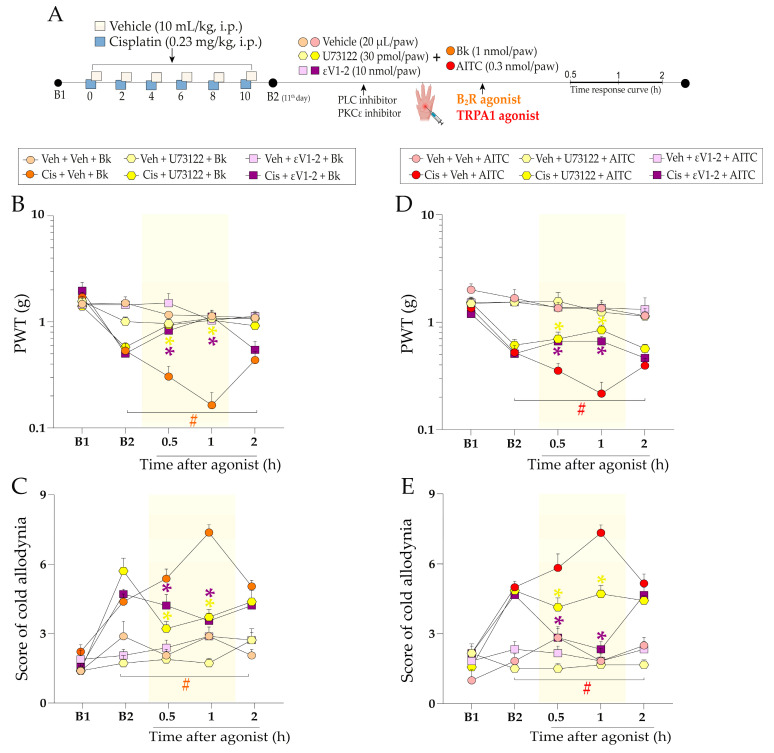
PLC and PKCε signalling pathways downstream from kinin B_2_ receptor activation contribute to cisplatin-induced painful behaviours. Cisplatin (Cis; 0.23 mg/kg) or vehicle (Veh; 10 mL/kg) were administered intraperitoneally (i.p.) in mice every 48 h for 10 days. On the 11th day, a sub-nociceptive dose of Bk (1 nmol/paw, i.pl.) or AITC (0.3 nmol/paw, i.pl.) was co-injected in mice via intraplantar (i.pl.) route with U73122 (30 pmol/paw, i.pl., PLC inhibitor) or εV1–2 (10 nmol/paw, i.pl., PKCε inhibitor) (**A**). Mechanical PWT (**B**,**D**) and cold allodynia (**C**,**E**) were evaluated from 0.5 h up to 2 h after co-injection of the inhibitors plus agonists. Baseline 1 (B1) values were assessed before cisplatin or vehicle administration. Baseline 2 (B2) values were measured on the 11th day after the first cisplatin or vehicle dose and before the treatments. The symbols on the bars indicate individual values for each animal. ^#^ *p* < 0.05 vs. vehicle plus AITC group. * *p* < 0.05 vs. cisplatin plus Bk/AITC group. Data are expressed as mean + SEM (n = 6–7/group) and were analysed using two-way ANOVA followed by the Bonferroni post-hoc test. PWT: paw withdrawal threshold. B_2_R: kinin B_2_ receptor.

Subsequently, we investigated the molecular signalling pathways downstream from the kinin B_2_ receptor activation in cisplatin-treated mice (Figure 7A). Local administration of PLC inhibitor (U73122, 30 pmol/paw, i.pl.) and PKCε inhibitor (εV1–2, 10 nmol/paw, i.pl.) attenuated the enhancement of Bk-evoked mechanical allodynia, with maximum inhibitions of 56 ± 8% and 61 ± 11% at 1 h after intraplantar Bk injection, respectively, (Figure 7B) (F (20, 120) = 8.47; *p* < 0.0001; Figure 7B). U73122 and εV1–2 also reduced the cold allodynia, with maximum inhibitions of 70 ± 6% and 74 ± 6% at 1 h after intraplantar Bk injection, respectively (Figure 7C) (F (20, 120) = 5.01; *p* < 0.0001; Figure 7C) in mice with cisplatin-induced peripheral neuropathy.

Hereafter, we evaluated whether signalling pathways downstream from the kinin B_2_ receptor activation could cooperate with TRPA1 channel activation in cisplatin-induced peripheral neuropathy. The inhibition of PLC and PKCε by U73122 and εV1–2, respectively, attenuated AITC-evoked responses on mechanical allodynia, with maximum inhibition of 55 ± 9% and 39 ± 6% at 1 h after intraplantar AITC injection, respectively, (Figure 7D) (F (20, 124) = 4.26; *p* < 0.0001; Figure 7D) and cold allodynia with maximum inhibition of 54 ± 5% and 96 ± 7% at 1 h after intraplantar AITC injection, respectively, in mice previously treated with cisplatin (Figure 7E) (F (20, 124) = 8.20; *p* < 0.0001; Figure 7E).

## 3. Discussion

CIPN is a significant adverse effect in cancer patients undergoing chemotherapy treatment regimens. This condition represents a severe healthcare concern that compromises oncology treatment and the life quality of patients and cancer survivors [11,15]. Thus, the need to better understand the underlying mechanisms of CIPN prevails. Our findings confirmed previous data on cisplatin-induced nociceptive behaviours (mechanical and cold allodynia) being mediated by the kinin receptors and TRPA1 channel and provided evidence of the interaction between kinin B_2_ receptors and TRPA1 channel in cisplatin-induced pain symptoms for the first time. In turn, using agonists and antagonists of kinin B_2_ receptors and the TRPA1 channel, we demonstrated the functional interaction between these receptors in the model of painful peripheral neuropathy induced by cisplatin. Lastly, we elucidated that the intracellular signalling pathways, phospholipase C (PLC) and protein kinase C epsilon (PKCε), downstream from kinin B_2_ receptor activation are critical to sensitising the TRPA1 channel in the cisplatin-induced peripheral neuropathy model in mice.

Clinically, cisplatin-treated patients manifest sensorial changes such as numbness, tingling, and pain in a shooting or burning form in symmetrical and distal areas, usually in feet and hands, but also in more proximal limb areas [8,37,38]. In the present study, mice treated with cisplatin developed mechanical allodynia and cold hypersensitivity, which were sustained for at least two weeks after the last cisplatin dose, consistent with a previous study [22]. Hypersensitivity in front of mechanical stimuli is a hallmark behavioural sign of CIPN observed in rodents [39,40,41,42,43,44], reflecting the allodynia phenotype in CIPN patients. This cisplatin dosage regimen also produced cold allodynia, another symptom associated with platinum-based anticancer drugs [5,16,24], and is in accordance with earlier reports of cold sensitivity in cisplatin-treated rodents [7,28,32].

Activation and sensitisation of nociceptors play a crucial role in painful behaviours following neurotoxic chemotherapy drug exposure. In this context, both B_2_ and B_1_ kinin receptors’ contribution to pain transduction has been widely demonstrated, including in chemotherapy-induced neuropathic pain models [19,20,45]. To strengthen our hypothesis, we extended these previous findings and confirmed that kinin B_2_ and B_1_ receptors contribute to the nociceptive behaviours induced by cisplatin.

In addition to kinin B_2_ and B_1_ receptors, TRPA1 channels also are involved in cisplatin-induced mechanical allodynia [24]. In preclinical models of CIPN, hyperalgesia/allodynia have been associated with a change in the expression and function of proteins essential to signal transduction, expressed in nociceptors, including TRP channels [11,23,46,47]. In this sense, Ta et al. (2009) showed an upregulation of TRPA1 and TRPV1 mRNA in trigeminal and dorsal root ganglia neurons following cisplatin exposure, reflecting the enhancement in nociceptor responsiveness and, consequently, the nociceptive behaviours observed in cisplatin-treated mice [47]. Strengthening the idea of the contribution of the TRPA1 channel to cisplatin-caused mechanical hypersensitivity, TRPA1-deficient mice presented notably reduced mechanical allodynia after cisplatin administration [24]. Here, we demonstrated that a TRPA1 antagonist attenuated cisplatin-induced mechanical and cold allodynia, reinforcing the TRPA1 channel’s involvement in the neurotoxic effect of cisplatin. Although the involvement of TRPA1 in mechanical allodynia has already been described, the effect of the TRPA1 on cisplatin-induced cold allodynia has not yet been evaluated. In this context, we showed the TRPA1 channel’s involvement in cisplatin-induced cold allodynia, which is consistent with the block of the TRPA1 channel, since this channel is a sensor for cold temperatures [34,48].

Once we confirmed the participation of the kinin B_2_ and B_1_ receptors and the TRPA1 channel in cisplatin-induced nociceptive behaviours, we verified the functional interaction between these receptors in this peripheral neuropathy model. For this, we injected a sub-nociceptive dose of kinin B_2_ (Bk) and B_1_ (DABk) receptor agonists or a TRPA1 channel (AITC) agonist in mice previously treated with cisplatin. The intraplantar injection with Bk and AITC intensified the mechanical nociception and cold sensitivity in mice previously treated with cisplatin without altering vehicle-treated animals’ mechanical and cold sensitivity. Our data also show that the enhancement in nociceptive behaviours induced by Bk and AITC in cisplatin-treated mice is mediated mainly via B_2_ receptors and the TRPA1 channel, respectively, since systemic pretreatment with antagonists selective for the kinin B_2_ receptor and TRPA1 channel inhibited the effects induced by its respective agonists. Similarly, hypersensitivity towards sub-nociceptive doses of TRPs and kinin B_2_ receptor agonists has been observed in different models of pain [19,49,50,51], including cisplatin-induced peripheral neuropathy [22]. Unlike that observed in a previous study [22], the sub-nociceptive dose of the B_1_ agonist DABk did not enhance mechanical and cold allodynia in animals previously treated with cisplatin. Although kinin B_1_ receptors are constitutively expressed in essential structures for the transduction of painful stimuli [28,29], the DABk sub-nociceptive dose could not sensitise the B_1_ receptor. These discrepancies could be due to the cisplatin dose used in both studies, as in the present study, we used a 10 times lower dose of cisplatin since it caused nociceptive behaviours similar to the previous study [22]. Thus, to avoid unnecessary suffering to the animals, the lowest cisplatin dose was chosen for this study and could be the primordial factor responsible for the absence of the sensitisation of the kinin B_1_ receptor by its agonist. Consequently, we did not use the B_1_ receptor agonist in the remainder of our study.

Together, these results provide additional data of the participation of the kinin B_2_ receptor and TRPA1 channel in painful symptoms induced by cisplatin. In this context, we supposed that activating the kinin B_2_ receptor could be essential to sensitising the TRPA1 channel in the cisplatin-induced peripheral neuropathy model. This putative hypothesis was raised since kinin receptors and the TRPA1 channel are expressed in the same nociceptive sensory neurons and contribute to pain stimulus transmission [25,27,28,35]. Although it has not been evaluated, cisplatin and Bk could activate the kinin B_2_ receptor expressed in mast cells [52,53]. Activation of mast cells would lead to the release of histamine and histaminergic receptor activation on sensory neurons inducing intracellular changes and, thus, TRPA1 channel activation [54].

Since TRP channels, such as TRPA1 can be activated indirectly by G protein-coupled receptors (GPCR) [33,35,55,56], we demonstrated that the pharmacological antagonism of the TRPA1 channel prevented the sensitizing effect of the kinin B_2_ receptor agonist in nociceptive behaviours in cisplatin-treated mice. Additionally, the sensitizing effect of the TRPA1 agonist on the mechanical and cold allodynia in cisplatin-treated mice was prevented by kinin B_2_ and B_1_ receptor antagonists. Therefore, our results suggest crosstalk between the kinin B_2_ receptor (and partial of B_1_ receptor) and TRPA1 channel in nociceptive transmission in this peripheral neuropathy model. This observation is reinforced by previous findings, demonstrating that the pharmacological antagonism or genetic deletion of the TRPA1 channel attenuates the mechanical hyperalgesia induced by kinin B_2_ and B_1_ receptor agonists [32,35,36].

TRPA1 acts as an integrator molecule and is a downstream target for specific nociceptive components such as bradykinin, triggering the amplification of nociceptive processes [34]. However, the signalling pathways that activate TRPA1 downstream of kinin receptors activation have not yet been elucidated in cisplatin-induced peripheral neuropathy. Kinin receptors are G protein-coupled, and their stimulation on the surface of nociceptive neurons leads to activation of the PLC pathway, resulting in the breaking of phosphatidylinositol 4,5-bisphosphate (PIP_2_) into diacylglycerol (DAG) and inositol triphosphate (IP_3_). IP_3_ promotes increased cytosolic calcium (Ca^2+^) levels, which, together with diacylglycerol, activate PKC [30,31,57]. Importantly, both PLC and PKC are implicated in sensitizing sensory neurons through modification of ion channel functionality, contributing to hyperalgesia and allodynia states in acute and chronic pain conditions [33,35,55,58,59,60]. Within the serine/threonine kinase family, the PKCε signalling role presents a particular contribution towards nociception and acts as a second messenger followed by the kinin receptors’ activation [56,61,62,63]. Thus, we investigated the involvement of PKCε and PLC signalling pathways, dependent on kinin B_2_ receptor activation in the cisplatin-induced peripheral neuropathy model.

Local administration of PLC and PKCε inhibitors significantly reduced the sensitizing effect in the mechanical and cold allodynia evoked by kinin B_2_ receptor selective agonist, demonstrating that the PLC/PKCε signalling cascade contributes to the sensitizing effect of kinin B_2_ receptor activation in cisplatin-treated mice. Our results align with previous data demonstrating that the PLC signalling pathways’ activation by kinin receptors is critical to hypersensitivity in different painful conditions in experimental animals [33,36,56,63].

Although the PLC activation is well established from GPCR stimulation [64], we observe a role of PLC and PKCε in the sensitizing effect on TRPA1 agonist-evoked pain symptoms in mice treated with cisplatin. This observation aligns with the previous data in which the inhibition of PLC and PKC attenuate TRPA1 activation in vitro and in vivo by TRPA1 agonist cinnamaldehyde [35,36]. Since PLC activation plays a significant role in regulating and activating TRP channels through PIP_2_ degradation, it is suggested that the TRPA1 activation may also positively modulate PLC activity by increased cytosolic Ca^2+^ levels [34,36].

As PLC and PKCε inhibitors attenuated the sensitizing effect induced by Bk and AITC, our data suggest that PLC/PKCε signalling pathways downstream of kinin B_2_ receptor activation may sensitise the TRPA1 channel [32,33,36,56]. Therefore, our data are in agreement with the literature, which shows that the GPCR activation, such as kinin B_2_ receptor and, consequently, the release of Ca^2+^ from intracellular stores and TRPA1 phosphorylation by PKC, could contribute to the indirect activation of this ion channel [32,34,36].

In summary, the interaction between the TRPA1 channel and kinin receptors suggests a novel mechanism in cisplatin-induced pain symptoms. Furthermore, the TRPA1 channel sensitisation through the kinin B_2_ receptor activation via PLC and PKCε seems involved in mechanical and cold hypersensitivity in the cisplatin-induced peripheral neuropathy model. Therefore, regulating the activation of signalling pathways downstream of the kinin B_2_ receptor’s activation could mitigate the painful peripheral neuropathy decurrent of the chemotherapy treatment.

## 4. Materials and Methods

### 4.1. Drugs and Reagents

Cisplatin (cis-diamminedichloridoplatinum II, C-Platin^®^; Blau, SP, Brazil), bradykinin (Bk; kinin B_2_ receptor agonist), DABk (kinin B_1_ receptor agonist), Icatibant (kinin B_2_ receptor antagonist), DALBk (kinin B_1_ receptor antagonist), allyl isothiocyanate (AITC; TRPA1 agonist), A967079 (A96; TRPA1 antagonist), U73122 (PLC inhibitor), and εV1–2 (PKCε inhibitor) were purchased from Sigma Chemical Company (St. Louis, MO, USA). Cisplatin and kinins B_1_ and B_2_ receptor antagonists were prepared in isotonic solution (0.9% NaCl). A967079 was dissolved in 10% DMSO and 5% Tween 80 in isotonic solution (0.9% NaCl). Phosphate-buffered saline (PBS; 10 mM) was used to dilute kinin B_1_ and B_2_ receptor agonists administered using the intraplantar route. TRPA1 agonist stock solution was dissolved in 10% DMSO in PBS. The stock solutions of U73122 were prepared in 10% absolute ethanol, while εV1–2 stock solutions were prepared in PBS. All the stock solutions were diluted to the desired concentration just before use. The final concentration of all solutions containing ethanol or DMSO did not exceed 0.5% and 1%, respectively, and did not cause any detectable effect per se. The control groups (vehicle) received the vehicles in which the treatments were solubilised. Oral and intraperitoneal treatments were administered in mice in a volume of 10 mL/kg, while intraplantar treatment did not exceed the volume of 20 µL per paw. The doses of the drugs used in this study were based on previous studies [19,22,56,65,66].

### 4.2. Animals

Adult male Swiss mice (25–30 g) were produced and provided by the Central Animal Facility from the Federal University of Santa Maria. The animals were kept under constant environmental conditions with a 12:12h light-dark cycle, ambient temperature (22 ± 2 °C), and relative humidity of 55 ± 10%. The animals were fed a standard pellet diet and allowed free access to water. The experimental protocols using animals were approved by the Institutional Animal Care and Use Committee of the Federal University of Santa Maria (processes #2120170222/2022 and #8563120922/2022) and were conducted according to the Animal Research: Reporting in vivo Experiments (ARRIVE) guidelines [67], the guidelines for investigation of experimental pain in conscious animals [68,69], and national and international legislation (guidelines of Brazilian Council of Animal Experimentation and the U.S. Public Health Service’s Policy on Humane Care and Use of Laboratory Animals). The intensities of noxious stimuli were the minimum necessary to demonstrate the consistent effects of the treatments. The group size for each experiment was based on studies with protocols similar to ours [22,51,56], which were confirmed using power calculations (G*Power version 3.1.9.7). Allocation concealment was performed using a randomisation procedure (http://www.randomizer.org/; accessed on 3 October 2022) and according to the baseline thresholds before and after the cisplatin administrations. All experiments were performed by experimenters blinded to the treatment conditions.

### 4.3. Peripheral Neuropathy Model Induced by Cisplatin

Cisplatin (0.23 mg/kg) was administered intraperitoneally (i.p.) every 48 hours (h) for 10 days (days 0, 2, 4, 6, 8, and 10), as previously described [22,70,71]. The animals were subjected to behaviour assessments on the 1st day after the complete peripheral neuropathy induction (i.e., on the 11th day after the first cisplatin or vehicle administration).

### 4.4. Study Design

#### 4.4.1. Characterisation of Cisplatin-Induced Painful Peripheral Neuropathy

Initially, we investigated whether cisplatin systemic treatment evokes mechanical and cold allodynia in mice. After evaluating the baseline paw withdrawal threshold (PWT) to mechanical stimulus and cold sensitivity of the mice, they were randomised into treatment groups. Mice received cisplatin in three different doses (0.023, 0.23, and 2.3 mg/kg, i.p.) or its vehicle (isotonic solution (0.9%), 10 mL/kg, i.p.) every 48 h for 10 days (days 0, 2, 4, 6, 8, and 10). The mechanical PWT was continuously evaluated 24 h after each cisplatin or vehicle administration up to 25 days after the first administration (following the protocol described below (4.5.1 Mechanical allodynia assessment)). Cold sensitivity was evaluated on days 5, 11, 18, and 25 after the first cisplatin or vehicle administration (following the protocol described below (4.5.2 Cold allodynia)). The experimental design is represented in Figure 1A.

#### 4.4.2. Kinin B_1_ and B_2_ Receptors and TRPA1 Channel Involvement in Cisplatin-Induced Painful Peripheral Neuropathy

To validate the involvement of kinin B_1_ and B_2_ receptors and TRPA1 channel on cisplatin-caused mechanical and cold allodynia in the peripheral neuropathy model, the mice received a single administration of kinin B_2_ (Icatibant; 100 nmol/kg, intraperitoneal, i.p.) or B_1_ (DALBk; 150 nmol/kg, i.p.) receptor antagonists or of the TRPA1 channel selective antagonist (A967079; 100 mg/kg, oral route, p.o.) or their vehicles (10 mL/kg, i.p. or p.o.) at 24 h after the last cisplatin dose (11th day). After treatments, the mechanical PWT and cold sensitivity were evaluated at different times (from 0.5 h up to 6 h). The experimental design is shown in Figure 2A.

We also investigated whether treatment with sub-nociceptive doses of agonists of the kinin B_2_ and B_1_ receptors and TRPA1 channel could enhance the nociceptive behaviours induced by cisplatin. For this, the animals were previously treated with cisplatin (0.23 mg/kg, i.p.) or vehicle (10 mL/kg, i.p.), and 24 h after the last cisplatin dose (11th day), the animals received an intraplantar (i.pl.) injection of low doses of kinin B_2_ (Bk; 1 nmol/paw) and B_1_ (DABk; 3 nmol/paw) receptor agonists and TRPA1 channel agonist (AITC; 0.3 nmol/paw) or their vehicles (20 µL/paw, i.pl.). Mechanical PWT and cold sensitivity were evaluated from 0.5 up to 2 h after injection of the agonist sub-nociceptive dose. The experimental design is shown in Figure 3A and Figure 4A.

In sequence to confirm the contribution of kinin B_2_ receptors and TRPA1 channel to the nociceptive behaviours, other animal groups were treated with kinin B_2_ receptor antagonist (Icatibant; 100 nmol/kg, i.p.) or with TRPA1 channel antagonist (A967079; 100 mg/kg, p.o.) at 24 h after the last cisplatin dose (11th day). After 0.5 h, the same animals received the sub-nociceptive doses of their respective agonists, Bk or AITC, via intraplantar injection. The mechanical PWT and cold sensitivity were assessed until treatments with the antagonists showed an effect. The experimental design is shown in Figure 3A and Figure 4A.

#### 4.4.3. Functional Interaction between Kinin B_2_ Receptor and TRPA1 Channel in Cisplatin-Induced Painful Peripheral Neuropathy

Next, we investigated whether the functional interaction between the kinin B_2_ receptor and TRPA1 channel might mediate the cisplatin-induced nociceptive behaviours. The animals were submitted to the experimental protocol of peripheral neuropathy induced with cisplatin. On the 11th day, the mice were treated with TRPA1 channel antagonist (A967079, 100 mg/kg, i.p.) or kinin B_2_ (Icatibant; 100 nmol/kg, i.p.) or B_1_ (DALBk; 150 nmol/kg, i.p.) receptor antagonists or. After 0.5 h of the treatment with A967079, the animals received an intraplantar injection of kinin B_2_ receptor agonist (Bk; 1 nmol/paw, i.pl.). The animals pre-treated with Icatibant or DALBk received an intraplantar injection of TRPA1 channel agonist (AITC; 0,3 nmol/paw, i.pl.). The mechanical PWT and cold sensitivity were assessed until treatments with the antagonists showed an effect. The experimental designs are shown in Figure 5A and Figure 6A.

#### 4.4.4. Intracellular Pathways Dependent on Kinin B_2_ Receptor Activation and TRPA1 Channel Sensitisation

We also investigated the involvement of intracellular signalling pathways mediated by PLC and PKCε in cisplatin-induced painful peripheral neuropathy model. For this, animals previously treated with cisplatin or vehicle received intraplantar co-injection containing either an inhibitor of PLC (U73122, 30 pmol/paw, i.pl.) or PKCε (εV1-2; 10 nmol/paw, i.pl.) plus kinin B_2_ receptor agonist (Bk, 1 nmol/paw, i.pl.) or TRPA1 channel agonist (AITC, 0.3 nmol/paw). Immediately after the intraplantar co-injection of agonists and inhibitors, the mechanical PWT and cold sensitivity were assessed until treatments with the inhibitors showed an effect. The experimental design is shown in Figure 7A.

### 4.5. Nociceptive Parameters

#### 4.5.1. Mechanical Allodynia Assessment

Cisplatin-induced mechanical allodynia was measured by using the up-and-down method [72,73]. Firstly, mice were placed individually in clear plexiglass boxes (7 × 9 × 11 cm) on elevated wire mesh platforms to allow access to the ventral surface of the hind paws to evaluate the mechanicalPWT. For behavioural accommodation, mice remained in the box for approximately 1.5 h before procedures. The mechanical PWT was determined before (basal measure) and after cisplatin administration with flexible nylon von Frey filaments. The mechanical PWT response, expressed in grams (g), was calculated from the resulting scores using von Frey filaments of different strengths (0.02–10 g) [73]. The development of mechanical allodynia was defined as a reduction in the PWT (g) compared with the vehicle group or baseline values (B1) before cisplatin administration.

#### 4.5.2. Cold Allodynia

Cold allodynia was evaluated in mice by using acetone-evoked evaporative cooling [51,74]. Mice were placed on a wire mesh floor, and a drop of acetone (20 µL) was applied three times on the plantar surface of the right hind paw. The behavioural response was analysed for 30 s and recorded in scores. The scores were 0 = no response; 1 = quick withdrawal, flick, or stamp of the paw; 2 = prolonged withdrawal or repeated paw flicking; 3 = repeated paw flicking with licking directed at the ventral side of the paw. The sum of the three scores was used for data analysis. Cold sensitivity was considered an increase in the nociceptive scores compared with the vehicle group or baseline values (B1) before cisplatin administration.

### 4.6. Statistical Analysis

Statistical analyses were carried out using Graph Pad Prism 8.0 software (Graph Pad, San Diego, CA, USA). To meet parametric assumptions, mechanical threshold data were log-transformed before analyses and data normality was confirmed using the Kolmogorov–Smirnov test. Results are expressed as mean + standard error of the mean (SEM). They were analysed using two-way analyses of variance (ANOVA) followed by the Bonferroni post-hoc test (time and treatment as factors; F values indicate the interaction between these factors, except for cold allodynia data in Figure 2C). *p*-values less than 0.05 (*p* < 0.05) were considered statistically significant.

## Data Availability

Data is contained in the article.
